# A Novel Artificial Intelligence–Enhanced Digital Network for Prehospital Emergency Support: Community Intervention Study

**DOI:** 10.2196/58177

**Published:** 2025-01-23

**Authors:** Ji Hoon Kim, Min Joung Kim, Hyeon Chang Kim, Ha Yan Kim, Ji Min Sung, Hyuk-Jae Chang

**Affiliations:** 1 Department of Emergency Medicine Yonsei University College of Medicine Seoul Republic of Korea; 2 Department of Preventative Medicine Yonsei University College of Medicine Seoul Republic of Korea; 3 Department of Biomedical System Informatics Yonsei University College of Medicine Seoul Republic of Korea; 4 Department of Data Business, Ontacthealth Seoul Republic of Korea; 5 Department of Cardiology Yonsei University College of Medicine Seoul Republic of Korea

**Keywords:** emergency patient transport, transport time, artificial intelligence, smartphone, mobile phone

## Abstract

**Background:**

Efficient emergency patient transport systems, which are crucial for delivering timely medical care to individuals in critical situations, face certain challenges. To address this, CONNECT-AI (CONnected Network for EMS Comprehensive Technical-Support using Artificial Intelligence), a novel digital platform, was introduced. This artificial intelligence (AI)–based network provides comprehensive technical support for the real-time sharing of medical information at the prehospital stage.

**Objective:**

This study aimed to evaluate the effectiveness of this system in reducing patient transport delays.

**Methods:**

The CONNECT-AI system provided 3 key AI services to prehospital care providers by collecting real-time patient data from the scene and hospital resource information, such as bed occupancy and the availability of emergency surgeries or procedures, using 5G communication technology and internet of things devices. These services included guidance on first aid, prediction of critically ill patients, and recommendation of the optimal transfer hospital. In addition, the platform offered emergency department medical staff real-time clinical information, including live video of patients during transport to the hospital. This community-based, nonrandomized controlled intervention study was designed to evaluate the effectiveness of the CONNECT-AI system in 2 regions of South Korea, each of which operated an intervention and a control period, each lasting 16 weeks. The impact of the system was assessed based on the proportion of patients experiencing transfer delays.

**Results:**

A total of 14,853 patients transported by public ambulance were finally selected for analysis. Overall, the median transport time was 10 (IQR 7-14) minutes in the intervention group and 9 (IQR 6-13) minutes in the control group. When comparing the incidence of transport time outliers (>75%), which was the primary outcome of this study, the rate was higher in the intervention group in region 1, but significantly reduced in region 2, with the overall outlier rate being higher in the intervention group (27.5%-29.7%, *P*=.04). However, for patients with fever or respiratory symptoms, the group using the system showed a statistically significant reduction in outlier cases (36.5%-30.1%, *P*=.01). For patients who received real-time acceptance signals from the hospital, the reduction in the percentage of 75% outliers was statistically significant compared with those without the system (27.5%-19.6%, *P*=.02). As a result of emergency department treatment, 1.5% of patients in the control group and 1.1% in the intervention group died (*P*=.14). In the system-guided optimal hospital transfer group, the mortality rate was significantly lower than in the control group (1.54%-0.64%, *P*=.01).

**Conclusions:**

The present digital emergency medical system platform offers a novel approach to enhancing emergency patient transport by leveraging AI, real-time information sharing, and decision support. While the system demonstrated improvements for certain patient groups facing transfer challenges, further research and modifications are necessary to fully realize its benefits in diverse health care contexts.

**Trial Registration:**

ClinicalTrials.gov NCT04829279; https://clinicaltrials.gov/study/NCT04829279

## Introduction

Emergency patient transport plays a critical role in providing timely and appropriate care to individuals in emergencies [1]. By swiftly and safely transporting patients to the nearest health care facility equipped to handle their specific needs, it helps to improve their chances of receiving timely medical intervention and contributes to better patient outcomes [2]. On the contrary, delays faced by the emergency medical services (EMS) in transport of patients to health care facilities can result in increased patient morbidity, mortality, and complications [3-5]. Ambulance diversion, a major factor delaying patient transport, is a practice in EMS where an ambulance is redirected from its original destination hospital to another medical facility [6,7]. Ambulance diversion typically occurs when the emergency department (ED) of a hospital is temporarily unable to accept new patients due to high patient volumes, lack of available beds, or other capacity constraints [8,9]. As the demand for medical services increased with the COVID-19 pandemic, ambulance diversions became more frequent [10,11]. Eventually, as cases of severely ill patients dying in ambulances without being able to get to the ED became more common in Korea, the country amended its Emergency Medical Services Act to prohibit emergency medical institutions from refusing to accept critically ill emergency patients.

Along with securing a transfer hospital as soon as possible, it is crucial to provide accurate information about the condition of the patient to the medical staff of the ED to which they will be transferred in order to provide optimal treatment [12-15]. In addition to the most basic clinical information such as their main symptoms, blood pressure, and state of consciousness, information on interventions performed by the prehospital care provider on-site or during transport (eg, oxygen administration, intravenous fluids, medications, or immobilization techniques) and time stamps (eg, time of the report, arrival at the site, and time at which treatment was performed) help the receiving ED medical staff to prepare for the arrival of the patient and deliver appropriate care [16,17]. However, in most prehospital phases, the transmission of patient information between prehospital care provider and ED medical staff mainly relies on telephones, and comprehensive information cannot be sufficiently delivered, leading to a potential risk of communication errors such as mishearing [18,19].

With the rapid development of digital information devices such as tablets and smartphones, various medical information systems are being introduced to EMS and emergency medical institutions [18-21]. These technologies allow prehospital care providers in the field to find hospitals with available resources and ED medical staff to receive patient information in real-time to provide medical services in continuity through mobile phones [18,19]. The use of these technologies is expected to enhance communication and data sharing between prehospital care providers and ED medical staff, ensuring a smoother transition of care and improving patient outcomes. However, the evidence is insufficient, and the process has not yet been widely commercialized.

We developed a new medical information system called CONnected Network for EMS Comprehensive Technical-support using Artificial Intelligence (CONNECT-AI). CONNECT-AI system used voice and image recognition technology for real-time sharing of information at the prehospital stage. It also included AI algorithms that supported first aid by prehospital care providers and the selection of transfer hospitals, as well as a platform that shared information and videos of patients being transported to the ED in real time. This study aimed to report on the development of the CONNECT-AI system and to evaluate its effectiveness in reducing transfer delays and facilitating the timely transfer of emergency patients. Our hypothesis was that the implementation of this system would decrease the occurrence of transport delays for emergency patients.

## Methods

### Study Setting

The study was conducted in 2 geographically adjacent areas, each under the jurisdiction of different local fire departments: the northwest region of Seoul (region 1) and Goyang City in Gyeonggi-do (region 2). The demographic characteristics and the composition of the EMS in these 2 regions are detailed in Table 1. A total of 5 fire stations and 9 EDs participated in this study. Prehospital care providers working in the NFA (National Fire Agency) assessed patients’ urgent conditions and, when necessary, performed a limited range of advanced care techniques in the prehospital setting. These techniques included administering intravenous fluids such as normal saline and glucose solution, placing advanced airways, and administering specific medications under the supervision of a medical director [22]. All patients assessed by prehospital care providers were transported to one of the EDs in accordance with the standard field first aid protocol developed by the NFA. This protocol included standardized first aid procedures and guidelines for selecting a hospital for transfer [23]. Prehospital care providers use their cell phones when they need to communicate with the medical staff of EDs to select a transfer hospital. During the study period, prehospital care providers were supposed to check for isolation unit availability at EDs to which they transfer patients with febrile or respiratory symptoms in response to COVID-19 pandemic. Prehospital care providers are required to manually record the prehospital run sheets (PHRS) managed by the NFA using tablet devices.

**Table 1:** Demographic characteristics and composition of the emergency medical system of the 2 regions.

Characteristics		Region 1	Region 2
Area (square miles)		27.5	110.5
Resident population (million), n		11	10
Fire station, n		3	2
Ambulance, n		20	17
**Emergency department, n (n)^a^**			
	Level 1	2 (2)	4 (4)
	Level 2	3 (3)	2 (0)

^a^The number in parentheses indicates the number of emergency departments that participated in this study.

### Study Design and Population

This community-based nonrandomized controlled intervention study with a blinded outcome assessment was designed to evaluate the effectiveness of the CONNECT-AI system for transporting patients by public ambulances in 2 communities (region 1 and region 2) that were selected as demonstration areas. The intervention group was defined as patients who were transported by public ambulance with an activated CONNECT-AI system, and the control group was defined as patients who were transported by public ambulance without an activated CONNECT-AI system (which is the same as the conventional practice). Public ambulances in region 1 underwent a 16-week intervention period beginning April 19, 2021, followed by a 1-week washout period and then a 16-week control period that ended on December 31. On the contrary, the public ambulances in region 2 had opposite intervention and control periods during the same time period.

During the study period, all patients transported by public ambulance in regions 1 and 2 to the 9 participating EDs were enrolled in the study. During the intervention period, all public ambulances in the regions were required to use the CONNECT-AI system, but if the situation was determined by prehospital care providers to be not appropriate for the use of the system, it was withdrawn, and the transported patients were excluded from the study. Patients were also excluded from enrollment if the ambulance had a special mission, such as transporting a person with COVID-19 infection or if the patient arrived at the ED but returned home or went to an outpatient clinic without treatment at the ED.

### System Development and Protocol (Intervention)

**Figure 1:**
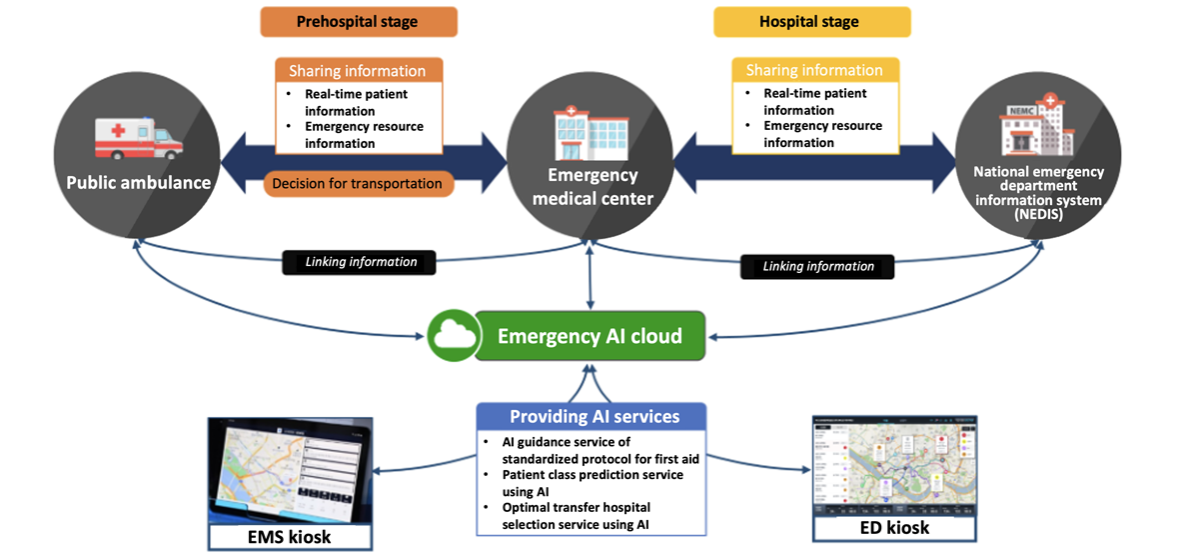
The overall architecture of the CONNECT-AI (CONnected Network for EMS Comprehensive Technical-Support using Artificial Intelligence) system. AI: artificial intelligence; ED: emergency department; EMS: emergency medical services.

The CONNECT-AI system captures all prehospital information in real-time through 5G communication technology and Internet of Things (IoT) devices. It provides 3 AI services on a cloud-based platform that can support the aids and decisions of prehospital care providers. The platform also enables medical staff in the ED to share real-time information in the prehospital setting before the ambulance arrives at the hospital. Furthermore, the present system can provide prehospital care providers with real-time information about ED crowding status and the availability of emergency medical resources at the hospital (Figure 1). This system collects patient information in real time from the transport scene during the prehospital stage using 5G communication technology and internet of things devices, while also gathering real-time data on emergency department crowding status and the availability of emergency medical resources from national emergency department information system. The collected information is processed through a cloud-based platform, providing 3 artificial intelligence (AI) services: standardized protocols for first aid, prediction of critical patients, and selection of the optimal hospital for transport. In addition, this platform enables real-time sharing of prehospital information with emergency department medical staff before the ambulance arrives at the hospital through emergency medical services kiosks and emergency department kiosks.

**Figure 2:**
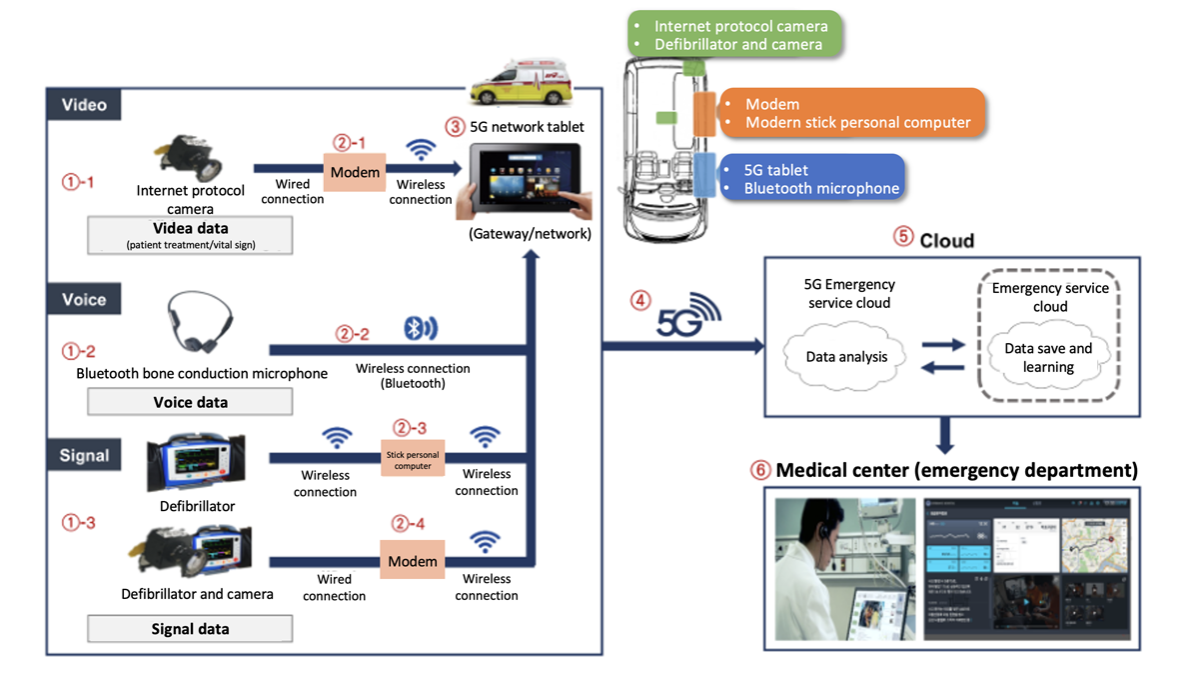
The flow of multimodal data from the public ambulance to the emergency department.

To use the CONNECT-AI system, a networking device that can collect and communicate video, voice, and vital sign information with the hospital in real time through a digital platform was installed in all ambulances; a schematic representation of this data flow is shown in Figure 2.

Real-time video, audio, and patient vital sign data, collected from a Bluetooth bone conduction microphone worn by prehospital providers and from a camera and defibrillator installed in the public ambulance, are transmitted to the medical staff in the emergency department by the 5G emergency service cloud.

Before the intervention period, all prehospital care providers were trained to use the CONNECT-AI system through a 1-hour in-person lecture with hands-on training. During the intervention period, prehospital care providers wore equipment (bone conduction microphones and EMS kiosks) to use the system, in contrast to the control period when they followed traditional practice. The following 3 AI assistant services have been developed to support prehospital care providers in performing first aid that was appropriate to the patient’s condition and to facilitate the transfer of the patient to the optimal hospital as quickly as possible: (1) AI guidance service of standardized protocol for first aid, (2) patient class prediction service using AI, and (3) optimal transfer hospital selection service using AI. Textbox 1 describes the details and purpose of the 3 AI assistant services as compared with the current prehospital practice without the CONNECT-AI system. Previous research has introduced the algorithm used in the optimal transfer hospital selection service [22].

**Three artificial intelligence (AI) assistant services for prehospital care providers.**

**AI guidance service of a standardized protocol for first aid**

- Service description: This service uses real-time patient information as an input to predict the treatment that should be performed on the patient at the hospital and outputs the results to the prehospital care providers.
- Current practice: Prehospital care providers use their experience and medical knowledge to assess patient severity.
- Expectancy of effects: Reduce the number of unpredictable, critically ill patients for inexperienced prehospital care providers.

**Patient class prediction service using AI**

- Service description: This service uses real-time patient information as input to predict the treatment that should be performed on the patient at the hospital and outputs the results to the prehospital care providers.
- Current practice: Prehospital care providers use their experience and medical knowledge to assess patient severity.
- Expectancy of effects: Reduce the number of unpredictable, critically ill patients for inexperienced prehospital care providers.

**Optimal transfer hospital selection service using AI**

- Service description: This service provides prehospital care providers with a real-time, prioritized list of hospitals that can provide predicted care, and allows for simultaneous requests for acceptance to multiple hospitals.
- Current practice: Prehospital care providers use their own judgment to determine the hospitals for transfer or use their cell phones to sequentially contact hospital medical staff to inquire about acceptance for transportation.
- Expectancy of effects: By using data communicated in real-time through the CONnected Network for EMS (emergency medical services) Comprehensive Technical-support using Artificial Intelligence system, prehospital care providers can quickly arrange the optimal hospital for a patient’s condition.

**Figure 3:**
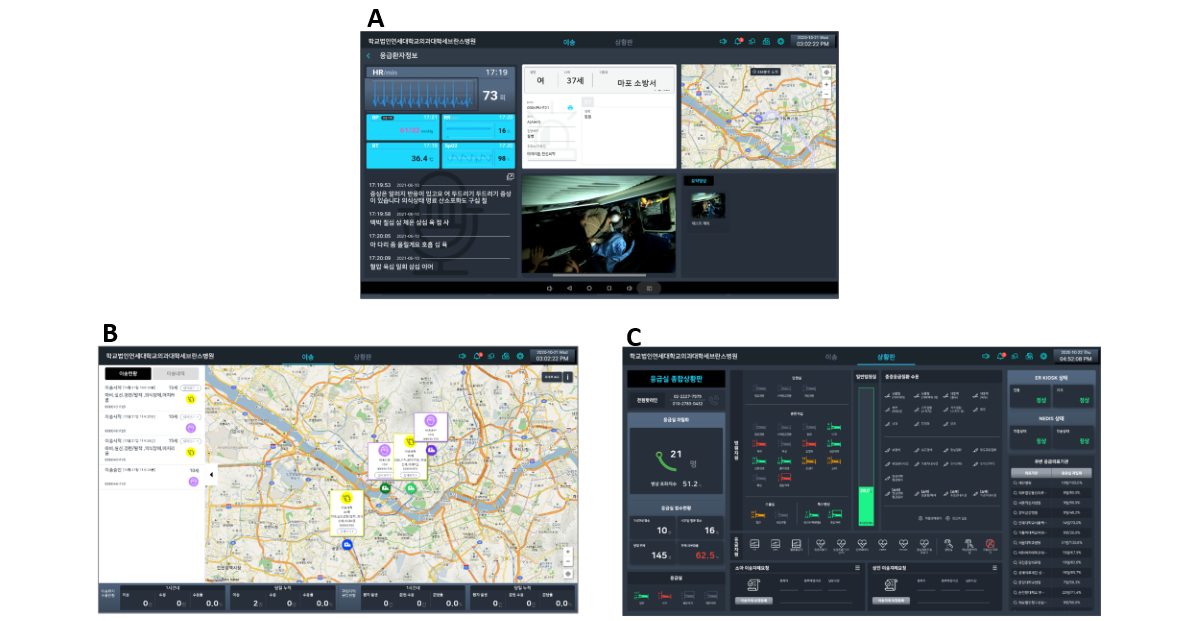
Main screens provided by the emergency department kiosk, (A) a screen that monitors a patient's real-time transport status and displays the predicted severity of the patient, (B) a screen that shows the public ambulances that are currently on their way to the hospital, and (C) a screen of real-time emergency resource information that be available in hospitals with kiosks installed.

The EDs in this study were equipped with kiosks to monitor the status of patients transported by public ambulance and to communicate with ambulances. This kiosk also allowed the physician in charge of the ED to monitor the real-time transport status of public ambulances enroute to the hospital, as well as information on available beds and emergency capacity in the ED (Figure 3).

The medical staff were provided with 1-hour hands-on training on how to communicate with prehospital care providers in real time through the kiosk. During the control period, prehospital care providers used their cell phones to communicate the status of patients requiring transport to ED medical staff and to inquire about patient acceptance sequentially as a traditional practice. In contrast, during the intervention period, the CONNECT-AI system provided prehospital care providers with a hospital list that was appropriate for the patient’s condition and allowed the patient to be transferred without additional contact if the ED medical staff did not send an unacceptable message on the system. In cases where prehospital care providers determined they needed to transport to EDs that showed that they could not accept the patient, providers clicked a button on the EMS kiosk to simultaneously send a request for acceptance to these EDs; the transport was then performed when they received an acceptance signal from the ED (Multimedia Appendix 1).

### Data Collection

In our study, prehospital stage information was obtained through the PHRS, which is electronically stored to provide basic prehospital operation information under the NFA. Data extracted from the PHRS included age, gender, past medical history, mental status, vital signs (blood pressure, pulse rate, respiratory rate, oxygen saturation, and body temperature), patient symptoms, and the first aid timeline. Mental status was assessed using the AVPU scale (A for awake, V for verbal response, P for pain response, and U for unresponsiveness). Hospital stage information was obtained from a dataset submitted by the participating medical institutions to the National Emergency Department Information System (NEDIS); this is a nationwide computerized system used to collect and analyze medical information of patients who visit EDs in South Korea, in accordance with the Act on Emergency Medical Care and managed according to the standardized protocol distributed by the National Emergency Medical Center [22]. Information on patient severity and ED treatment outcomes for those transported by public ambulance was extracted from the NEDIS. Patient severity was assessed using the Korean Triage and Acuity Scale (KTAS), a classification system used in EDs across Korea. The KTAS, derived from the Canadian Triage and Acuity Scale, is a 5-level classification system where 1 indicates resuscitation and 5 represents nonemergent cases [24]. Based on the linkage of these 2 main datasets, log records of events that occurred when prehospital care providers used the system during the intervention period were added by extracting them from a cloud-based digital platform.

### Outcome

The primary outcome of our study is the proportion of outliers with long transport times among the transported patients. Transport time is defined as the period from the time the prehospital care provider left the scene to the time of arrival at the hospital. Patient mortality was defined as the death of a patient who was alive upon arrival but died during treatment in the ED.

### Sample Size Calculation

In 2018, the mean of transport times for one year were 21 (SD 10) minutes for region 1, 16 (SD 8) minutes for region 2, and 20 (SD 25) minutes overall. Based on these figures, the primary outcome of this study, transport time, was assumed to show an improvement of more than 10% (a 2-minute difference) when using the CONNECT AI system, compared with the conventional ambulance transport standard. The largest standard deviation of 25 minutes was conservatively chosen for calculations, and it was predicted that a statistical power of over 90% could be achieved. Since transport time is a continuous variable, the sample size was calculated based on a 2-sample *t* test, resulting in an estimate of approximately 10,000 cases. A proposal was made to the regional administrative authorities for a pilot period during which 10,000 transports were expected to occur, and this proposal was accepted by the regional administrative authorities.

### Statistical Analysis

Continuous variables were expressed as mean values with standard deviations and compared between groups using the Student *t* test or Mann-Whitney *U* test. Categorical variables were expressed as frequencies and percentages and compared using the chi-square test. All tests were 2-sided, with a statistical significance of *P*<.05. All statistical analyses were performed using SAS software (version 9.4; SAS Institute).

### Ethical Considerations

This community-based interventional study was performed in accordance with the revised Declaration of Helsinki and was approved by the institutional review board of Severance Hospital, South Korea on April 14, 2021 (approval number 4-2021-0217). This research was prospectively registered on ClinicalTrials.gov (NCT04829279). As the patient information covered by this study was within the scope of being provided by the NFA and the National Emergency Medical Center (NEMC) under the Ministry of Health and Welfare with personally identifiable information removed based on the law, informed consent from the patients included in the study was waived. However, as audio and video data of the prehospital care providers were collected and processed during the intervention, written informed consent for the use of this information was obtained from them voluntarily before the start of the intervention, and no material and financial compensation was provided for participation in the study.

## Results

### Population Characteristics

**Figure 4:**
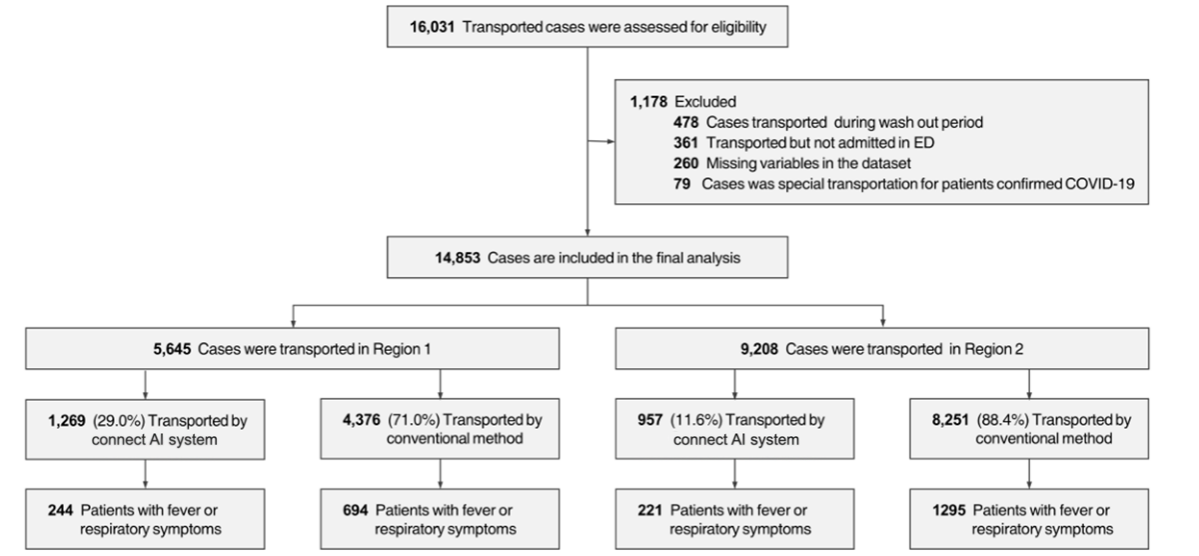
Patient enrollment flow. AI: artificial intelligence; ED: emergency department.

During the study period, there were a total of 16,031 patients transported by public ambulance who met the inclusion criteria. After excluding patients transferred during the washout period and those eligible for exclusion, 14,853 patients were finally selected for analysis. Of them, 1269 (29.0%) and 957 (11.6%) patients were included in the intervention group from regions 1 and 2, respectively (Figure 4). The baseline characteristics of the intervention and control groups are presented in Table 2. The proportion of the intervention group enrolled was higher in region 1 than in region 2. Febrile and respiratory symptoms were more frequent in the intervention group than in the control group.

**Table 2:** Baseline characteristics.

Characteristics				Total								Region 1												Region 2							
				Control (n=12,627)		Intervention (n=2226)		*P* value			Control (n=8251)			Intervention (n=957)			*P* value				Control (n=4376)					Intervention (n=1269)			*P* value		
Age (years), mean (SD)				56.88 (23.38)		58.00 (23.65)		.04			57.98 (22.87)			60.45 (23.13)			.002				54.81 (24.17)					56.15 (23.88)			.08		
Sex (male), n (%)				6635 (52.55)		1155 (51.89)		.60			4218 (51.12)			533 (55.69)			.007				2417 (55.23)					622 (49.01)			<.001		
**KTAS^a^ score, n (%)**									<0.001										.004												<.001
	1		467 (3.70)		74 (3.32)					288 (3.49)			28 (2.93)							179 (4.09)					46 (3.62)						
	2		1187 (9.40)		276 (12.40)					837 (10.14)			118 (12.33)							350 (8.00)					158 (12.45)						
	3		5267 (41.71)		962 (43.22)					3547 (42.99)			396 (41.38)							1720 (39.31)					566 (44.60)						
	4		5048 (39.98)		785 (35.27)					3115 (37.75)			338 (352.32)							1933 (44.17)					447 (35.22)						
	5		658 (5.21)		129 (5.80)					464 (5.62)			77 (8.05)							194 (4.43)					52 (4.10)						
Fever or respiratory symptoms, n (%)				1989 (15.75)		465 (20.89)		<.001			1295 (15.7)			221 (23.09)			<.001				694 (15.86)					244 (19.26)			.005		
Systolic blood pressure, mean (SD)				129.01 (40.41)		129.11 (40.83)		.91			131.43 (39.51)			134.49 (36.62)			.01				124.44 (41.68)					125.06 (43.31)			.64		
Diastolic blood pressure, mean (SD)				75.03 (23.9)		74.09 (23.33)		.09			75.81 (23.61)			75.96 (21.67)			.84				73.56 (24.37)					72.68 (24.42)			.25		
Heart rate, mean (SD)				81.78 (32.56)		81.87 (25.33)		.89			82.59 (33.96)			82.95 (21.91)			.66				80.25 (29.67)					81.05 (27.61)			.37		
Respiratory rate, mean (SD)				17.90 (5.46)		17.95 (5.22)		.68			18.42 (5.42)			18.84 (4.34)			.01				16.91 (5.39)					17.27 (5.7)			.04		
Oxygen saturation, mean (SD)				37.16 (48.58)		56.30 (52.40)		<.001			23.89 (42.60)			28.30 (54.45)			.02				61.88 (49.37)					77.13 (39.60)			<.001		
Body temperature, mean (SD)				36.05 (6.43)		36.38 5.85)		.01			36.12 (5.37)			36.27 (4.44)			.32				35.92 (8.05)					36.46 (6.72)			.01		
**Mental status^b^, n (%)**									.01										.04												.12
	Awake		11,615 (92.06)		2041 (92.27)					7614 (92.3)			879 (91.95)							4001 (91.6)					1162 (92.52)						
	Verbal response		409 (3.24)		83 (3.75)					225 (2.73)			32 (3.35)							184 (4.21)					51 (4.06)						
	Pain response		279 (2.21)		56 (2.53)					214 (2.59)			33 (3.45)							65 (1.49)					23 (1.83)						
	Unresponsiveness		314 (2.49)		32 (1.45)					196 (2.38)			12 (1.26)							118 (2.7)					20 (1.59)						
**Fire stations, n (%)**									<.001									<.001												<.001	
		1	3490 (27.64)		62 (2.79)					3490 (42.3)			62 (6.48)							—^c^					—^c^						
		2	4761 (37.70)		895 (40.21)					4761 (57.70)			895 (93.52)							—^c^					—^c^						
		3	1277 (10.11)		702 (31.54)					—^c^			—^c^							1277 (29.18)					702 (55.32)						
		4	1637 (12.96)		378 (16.98)					—^c^			—^c^							1637 (37.41)					378 (29.79)						
		5	1462 (11.58)		189 (8.49)					—^c^			—^c^							1462 (33.41)					189 (14.89)						
**ED^d^ result, n (%)**									.01										.32												.12
		Admission	3583 (28.59)		687 (30.92)					2358 (28.74)			287 (30.02)							1225 (28.30)					400 (31.60)						
		Discharge	8549 (68.21)		1461 (65.75)					5630 (68.61)			644 (67.36)							2919 (67.44)					817 (64.53)						
		Transfer	208 (1.66)		49 (2.21)					92 (1.12)			15 (1.57)							116 (2.68)					34 (2.69)						
		Expire	194 (1.55)		25 (1.13)					126 (1.54)			10 (1.05)							68 (1.57)					15 (1.18)						

^a^KTAS: Korean Triage and Acuity Scale. The KTAS was evaluated from 1, resuscitation, to 5, nonemergency.

^b^Mental status was assessed using the AVPU scale.

^c^Not applicable.

^d^ED: emergency department.

The median transport time was significantly longer for the intervention group (10, IQR 7-14 min) with the system activated than for the control group without the system (9, IQR 6-13 min). For a comprehensive analysis of the study results, we presented a table showing the comparison of transport times as a continuous variable (Multimedia Appendix 2). In addition, to determine whether delays were caused by prehospital care providers struggling to adapt to the new system, we analyzed trends in weekly median transport times throughout the entire intervention period and found no delays related to adaptation (Multimedia Appendix 3).

### Proportion of Outliers With Longer Transport Times According to the Intervention

**Figure 5:**
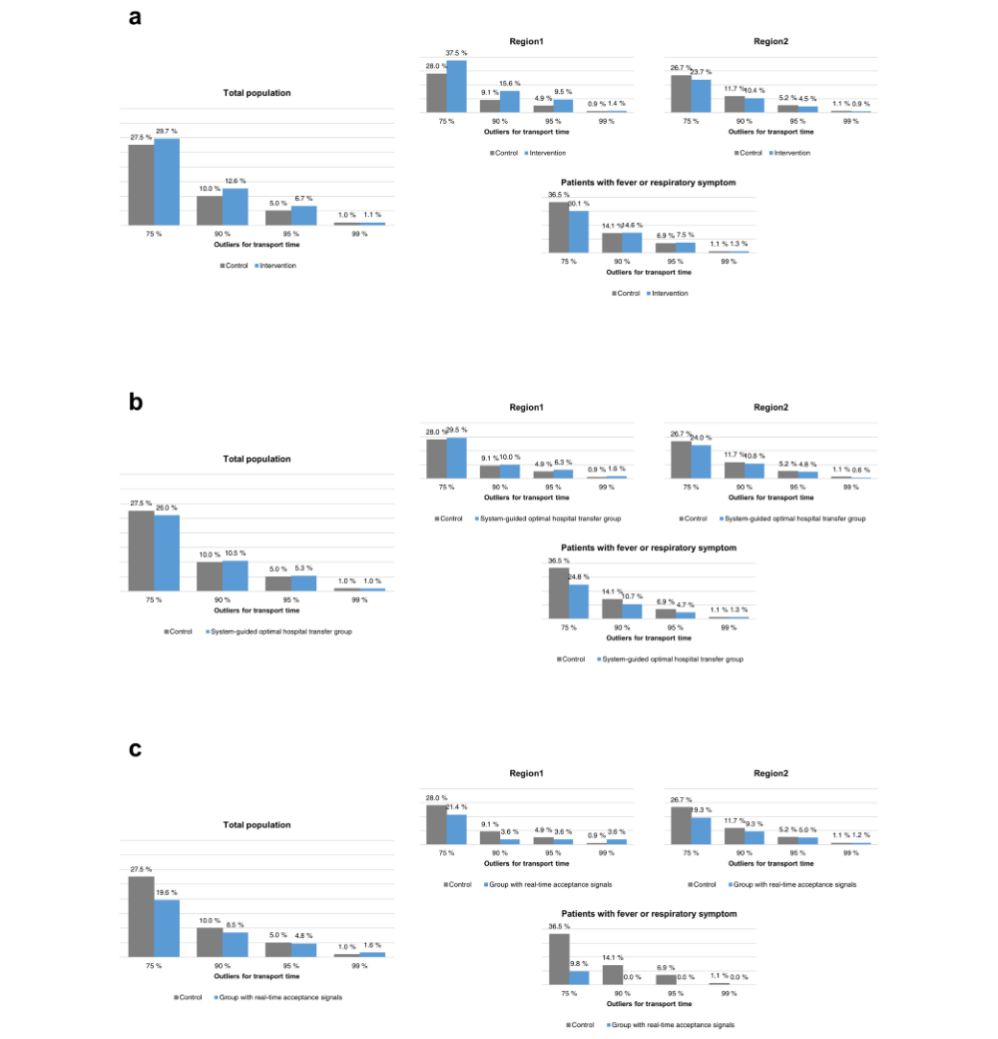
The proportion of outliers with longer transport times according to the intervention.

Figure 5 shows the difference in the proportion of outliers with longer transport times according to the intervention. In the overall population and region 1, a higher proportion of outliers in transport time occurred in the intervention group. On the contrary, region 2 showed a significant reduction in the proportion of cases with a percentile transport time greater than 75% (26.7%-23.7%, *P*=.05). For patients with fever or respiratory symptoms, there was a statistically significant reduction of more than 75% in the proportion of outlier cases in the group that used the system (36.5%-30.1%, *P*=.01).

In the system-guided optimal hospital transfer group, there was no significant difference in outlier proportions, compared with the control group; however, for patients with fever or respiratory symptoms, a significant reduction was noted in outliers greater than 75% (36.5%-24.8%, *P*<.001).

For patients who received real-time acceptance signals from the hospital, the proportion of outliers was reduced, compared with the group without the system, and the 75% reduction in the percentage of outliers was statistically significant (27.5%-19.6%, *P*=.01). For patients with fever or respiratory symptoms, the group that transferred after receiving the acceptance signal had no 90% outliers for transport time.

### Patient Mortality in the ED

Multimedia Appendix 4 shows the proportion for deaths after arrival at the ED according to the intervention. The mortality rate in the ED was 1.5% in the control group and 1.1% in the intervention group (*P*=.14). In the system-guided optimal hospital transfer group, the proportion for deaths after arrival at the ED was significantly smaller than the control group (1.54%-0.64%, *P*=.01).

A total of 114 prehospital care providers who participated in the study were surveyed about their satisfaction with three AI assistant services. The optimal transfer hospital selection service using AI was found to be the most satisfactory for prehospital care providers (Multimedia Appendix 5).

## Discussion

### Principal Results

As a result of applying this system, there was no significant change in the overall proportion of delayed patients and patient transport time. The median time required for patient transfer in this study was 9-10 minutes, which is extremely short when compared with that from previous studies in other countries [18,19]. In Korea, as the EMS system is exclusively operated by the NFA with government funds, patients are not obligated to pay for transportation, and public ambulances are relatively accessible compared with other countries [25]. High accessibility to public ambulances was accompanied by the side effect that even patients with low case severity frequently use public ambulances. Likewise, in this study, it was confirmed that mild patients with KTAS 4 and 5 accounted for 44.6% of all the transferred patients. Patients with low severity have a wider choice of hospitals compared with severe emergency patients; therefore, the risk of delay in transfer is low, even without using the CONNECT-AI system. This explains why the effectiveness of CONNECT-AI system was dominated by specific groups, such as patients with fever or respiratory symptoms who had difficulty selecting a transfer hospital, rather than a reduction in the proportion of outliers with long transport times among all the transferred patients. In this subgroup, it was confirmed that the transfer delay was noticeably reduced when the hospital responded to transfer acceptance through the ER kiosk.

In our study, compared with the control group, the rates of system usage by prehospital care providers in the intervention group were 29.0% and 11.6% for regions 1 and 2, respectively, considerably low. As this system was not unified with the existing EMS system, it depended on the prehospital care provider’s decision whether or not to use our system. As prehospital care providers were not obligated to use this system, it was used more frequently on patients with fever and respiratory symptoms who had difficulty obtaining transfer permission from the hospital than on patients who had no difficulty in hospital assignment.

According to the results of a survey conducted on users who used this new medical system, the function that prehospital care providers were most satisfied with was the role of the EMS kiosk as a platform to easily obtain information on crowding in the ED, particularly because the EMS kiosk can contact multiple hospitals simultaneously and help to find a transfer hospital more quickly. However, in this study, the number of cases in which the medical staff responded with an acceptable answer to the transfer request by the prehospital care provider was insufficient to demonstrate the effectiveness of this system in reducing transport time. We hypothesize that this situation can be attributed to 2 primary reasons. First, ED medical staff faced challenges in promptly addressing requests from prehospital care providers due to their busy engagement in providing emergency treatment to existing patients within the ED. The second reason is the challenging nature of the ED, which lacks control over patient input and is susceptible to overcrowding, rendering it difficult for medical staff to actively decide on accepting new patients [26].

To provide the best emergency care to more patients with limited medical resources in the region, it is not only important to transfer each patient promptly, but emergency patients must also be properly distributed to each hospital [27-29]. The AI prediction algorithm for selecting the optimal transfer hospital of this system contains a strategy to guide patients, who need resuscitation or emergency procedures, to hospitals with sufficient resources and patients with mild symptoms to small hospitals. Such a resource-saving strategy is expected to be advantageous for more efficient use of limited resources but cannot be an absolute solution in a situation where the demand of patients exceeds the supply of medical resources. To balance the demand of patients and the supply of medical resources in a region, a system that leads to policy decisions on the supply of additional emergency medical resources is needed through real-time monitoring of the overall status of resource use and prediction of impending resource depletion [30,31]. We anticipate that real-time information on patient transport and hospital acceptance collected through our EMS kiosk and ED kiosk will be used as valuable data in these emergency medical resource management systems in the future.

To globally expand our initiative, several challenges must be addressed. First, discussions need to take place regarding existing legal restrictions and concerns about privacy and security when transmitting patient information over networks, as the legal framework varies by country. In addition, legal issues surrounding telemedicine must be resolved in advance [32,33]. Furthermore, many underserved and rural areas may lack access to networks capable of real-time communication, making the establishment of broader network infrastructure a priority over specific technologies.

### Comparison With Previous Works

In this study, a prehospital clinical decision support system and a patient information-sharing system during transport were developed. To the best of our knowledge, this AI-enhanced digital network system is the first clinical decision support system that determines the transfer hospital by comprehensively considering a wide range of information, including the clinical characteristics of the patient and the hospital resource information, at the prehospital stage. Hospital resource information such as ED crowding and the availability of emergency interventions is an important factor for hospital medical staff to provide the best care to patients, but it is not easy for prehospital care providers to verify hospital information and consider the same when selecting a transfer hospital. This system included machine learning algorithms that can effectively use vast amount of information. In recent years, AI systems that support decision-making have been widely introduced in the medical field but are rarely found in the EMS domain [34-36]. This is because data input is required to operate the AI algorithm, but real-time data input is almost impossible from the site of rescuing emergency patients [37]. Accordingly, voice- and image recognition technologies were applied to use information from the scene in real-time. This clinical information of the patient being transported was transmitted to the ER kiosk so that the ED medical staff could check the condition of the patient before arrival and prepare for emergency treatment.

### Limitations

This study has a few limitations. First, the number of patients who used this system was extremely small to sufficiently confirm the effect of the system. The cumbersomeness of having to use additional equipment such as an EMS kiosk and a microphone in addition to the existing tablet made prehospital care providers reluctant to use it. The proportion of patients with fever or respiratory symptom was higher in the group where the system was used compared with the control group. This could be explained by the prehospital providers’ assessment that the potential benefits of using the system outweighed the inconvenience, especially when the transport was more challenging. To address the resulting selection bias, we separated the group of patients with fever or respiratory symptom and confirmed that the system’s effectiveness was more pronounced in cases where transport difficulty was greater. Second, as this study did not cover patients transferred to hospitals outside the study area or patients transferred from other areas to hospitals in the study area, a comprehensive analysis of patient transfers within the area was not possible. The effectiveness of this system may be affected by the availability of medical resources across the region and therefore needs to be evaluated at the regional level. Including these patients in the study likely led to longer transport times for the outliers, as the transport distances were greater compared with transfers within the local area. Finally, in Korea, prehospital care providers receive permission from hospital medical staff to transport patients, but they can be transported without permission. Regulations on emergency patient transport may vary with the country; therefore, this aspect needs to be considered when understanding our system and interpreting the study results. Given that the intervention effect was more pronounced in the group with real-time acceptance signals in our study, it is likely that the system’s effectiveness was underestimated in this study setting compared with regions where transport authorization is mandatory.

### Conclusions

This digital platform offers a novel approach to enhancing emergency patient transport by leveraging AI, real-time information sharing, and decision support. While the system demonstrated improvements for certain patient groups facing transfer challenges, further research and adaptations are necessary to fully realize its benefits in diverse health care contexts.

## Appendix

Multimedia Appendix 1 Video clip introducing CONNECT AI (CONnected Network for EMS Comprehensive Technical-Support using Artificial Intelligence) system.

Multimedia Appendix 2 Supplementary Table 1. Comparison of the transport time between the two groups.

Multimedia Appendix 3 Supplementary Figure 1. The median transport time on a weekly basis during the intervention period. No delays related to the adaptation of pre-hospital care providers were observed in the early phase of the intervention period.

Multimedia Appendix 4 Patient mortality in the emergency department.

Multimedia Appendix 5 CONNECT AI system satisfaction survey.

## Abbreviations

AI: artificial intelligence
CONNECT-AI: CONnected Network for EMS Comprehensive Technical-Support using Artificial Intelligence
ED: emergency department
EMS: emergency medical services
IoT: Internet of Things
KTAS: Korean Triage and Acuity Scale
NEDIS: National Emergency Department Information System
NEMC: National Emergency Medical Center
NFA: National Fire Agency
PHRS: prehospital run sheets

## Data Availability

The datasets generated and analyzed during this study are available from the corresponding author on reasonable request.
