# Supplementary material for: A Novel Artificial Intelligence–Enhanced Digital Network for Prehospital Emergency Support: Community Intervention Study
Source: J Med Internet Res. 2025 Jan 23;27:e58177. doi: 10.2196/58177 (PMC11803323; doi:10.2196/58177)
Supplement: Multimedia Appendix 2 [file jmir_v27i1e58177_app2.docx]

Supplementary Table 1. Comparison of the transport time between the two groups

|  | Total | | | Region 1 | | | Region 2 | | |
| --- | --- | --- | --- | --- | --- | --- | --- | --- | --- |
|  | Control | Intervention | p-value | Control | Intervention | p-value | Control | Intervention | p-value |
| All patients | 9 (6-13) | 10 (7-14) | <0.001 | 9 (5-13) | 10 (6-15) | <0.001 | 10 (7-14) | 10 (7-13) | 0.578 |
| Subgroup |  |  |  |  |  |  |  |  |  |
| KTAS1 | 8 (5-11) | 8 (6-13) | 0.337 | 7 (4-11) | 12.5 (7.5-15.5) | <0.001 | 9 (6-12) | 7 (5-10) | 0.010 |
| KTAS2 | 9 (6-14) | 10 (7-14) | 0.132 | 9 (5-13) | 10 (6-15) | 0.053 | 10 (7-14) | 10 (7-13) | 0.380 |
| KTAS3 | 9 (6-14) | 10 (7-15) | <0.001 | 9 (6-14) | 11 (7-16) | <0.001 | 10 (7-15) | 10 (7-14) | 0.114 |
| KTAS4 | 9 (6-13) | 10 (7-14) | 0.003 | 9 (5-13) | 10 (6-15) | <0.001 | 10 (7-15) | 10 (7-13) | 0.228 |
| KTAS5 | 8.5 (5-13) | 8 (5-12) | 0.394 | 8 (5-12) | 7 (5-12) | 0.395 | 10 (7-13) | 9 (6-11.5) | 0.377 |
| Fever or respiratory symptoms | 10 (7-15) | 10 (7-14) | 0.353 | 10 (6-15) | 11 (6-16) | 0.132 | 11 (8-17) | 10 (7-13) | 0.001 |
| Other symptoms | 9 (6-13) | 10 (7-14) | <0.001 | 9 (5-13) | 10 (6-15) | <0.001 | 10 (6-14) | 10 (7-14) | 0.600 |
