# Supplementary material for: A Novel Artificial Intelligence–Enhanced Digital Network for Prehospital Emergency Support: Community Intervention Study
Source: J Med Internet Res. 2025 Jan 23;27:e58177. doi: 10.2196/58177 (PMC11803323; doi:10.2196/58177)
Supplement: Multimedia Appendix 3 [file jmir_v27i1e58177_app3.docx]

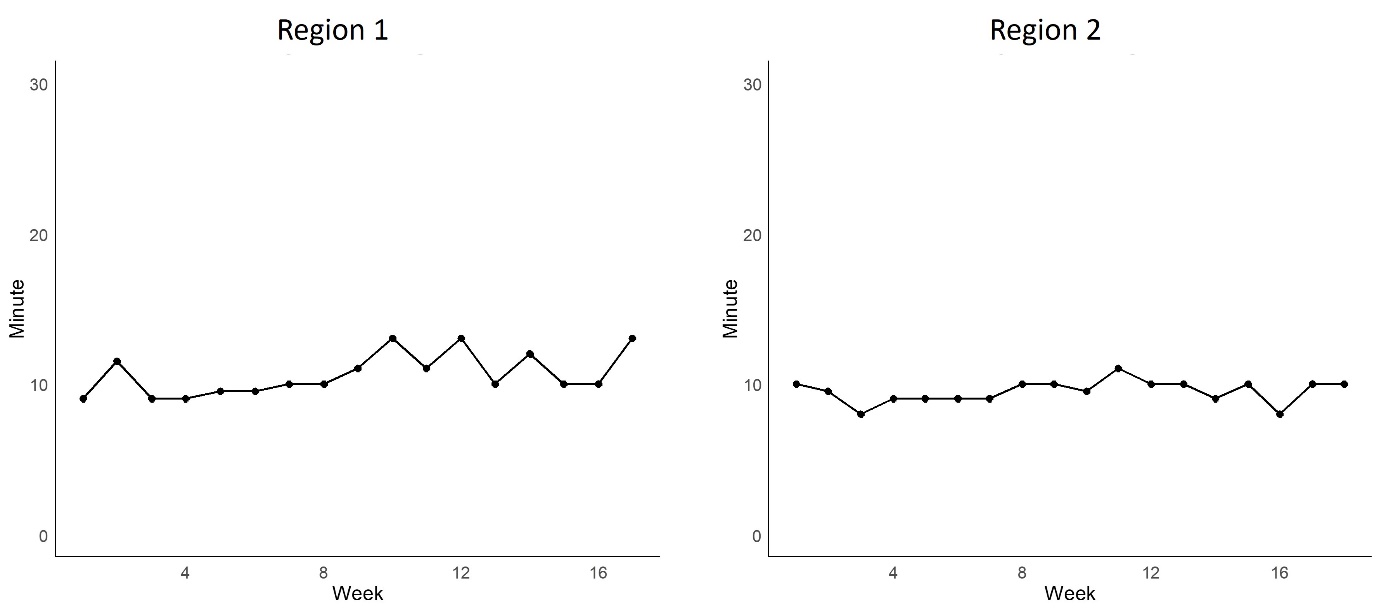


Supplementary Figure 1. The median transport time on a weekly basis during the intervention period. No delays related to the adaptation of pre-hospital care providers were observed in the early phase of the intervention period.
