# Supplementary figures and images for: A Novel Artificial Intelligence–Enhanced Digital Network for Prehospital Emergency Support: Community Intervention Study
Source: J Med Internet Res. 2025 Jan 23;27:e58177. doi: 10.2196/58177 (PMC11803323; doi:10.2196/58177)

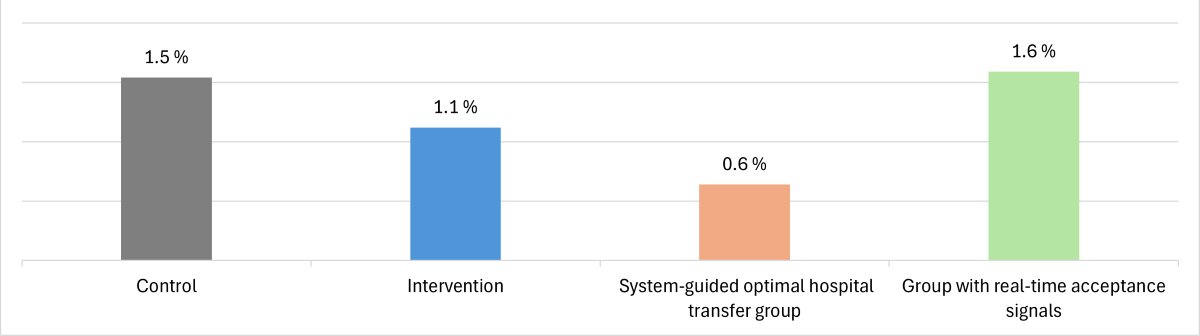

Supplement: Multimedia Appendix 4 [file jmir_v27i1e58177_app4.png]
