# Supplementary material for: A Novel Artificial Intelligence–Enhanced Digital Network for Prehospital Emergency Support: Community Intervention Study
Source: J Med Internet Res. 2025 Jan 23;27:e58177. doi: 10.2196/58177 (PMC11803323; doi:10.2196/58177)
Supplement: Multimedia Appendix 5 [file jmir_v27i1e58177_app5.docx]

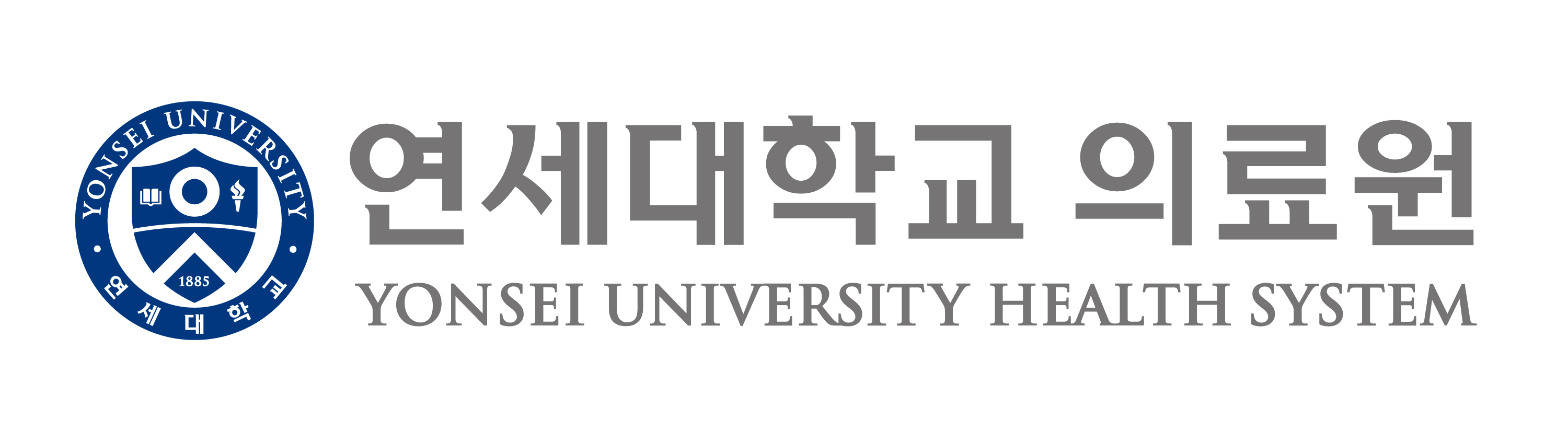

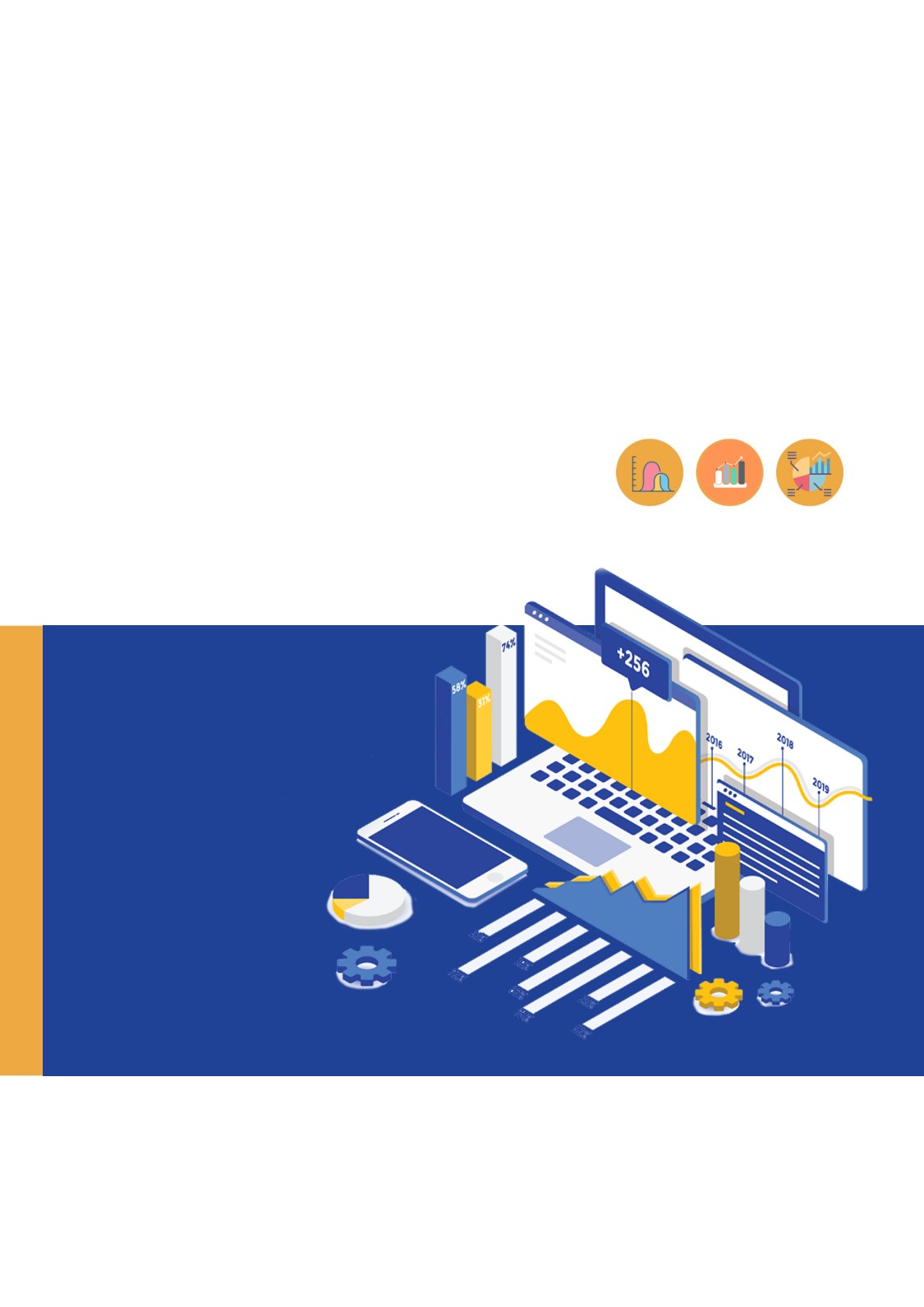


**CONNECT AI system satisfaction survey**

**- Final report-**


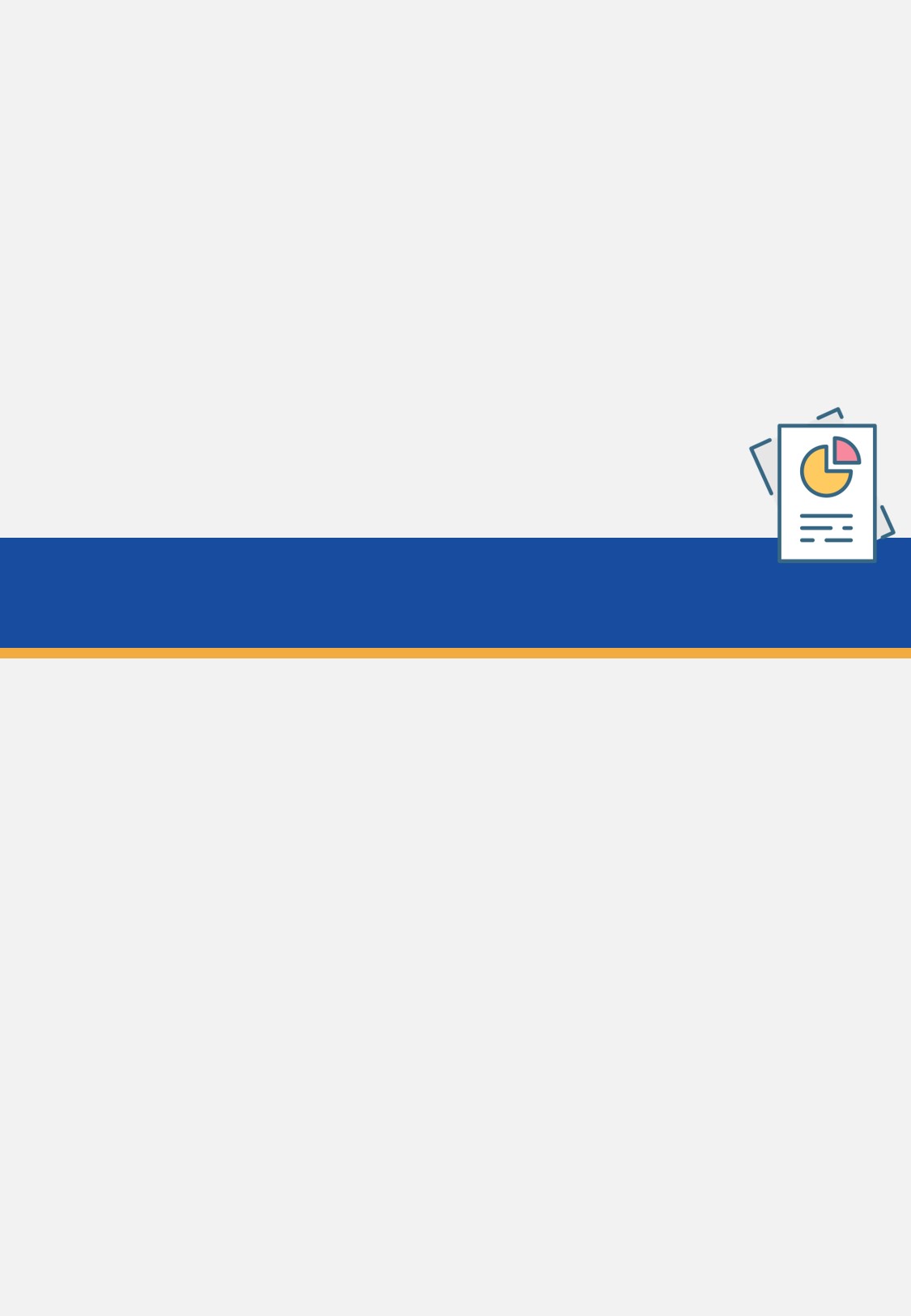


**Survey Results**

1. AI Guidance Service of Standardized protocol for first aid

2. Patient class prediction service using AI

3. Optimal Transfer Hospital Selection Service using AI 1. Survey


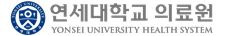


|  |  |
| --- | --- |

1. AI Guidance Service of Standardized protocol for first aid

|  | ※ AI Guidance Service of Standardized protocol for first aid  - The EMS kiosk presents standardized first aid for pre-hospital providers to perform based on patient information entered in real-time through the CONNECT AI system. |
| --- | --- |

[ figure 1] AI Guidance Service of Standardized protocol for first aid

|  | |  |  | |
| --- | --- | --- | --- | --- |
|  |  |  |  |  |
|  |  | (N=114) |  |  |
|  |  | 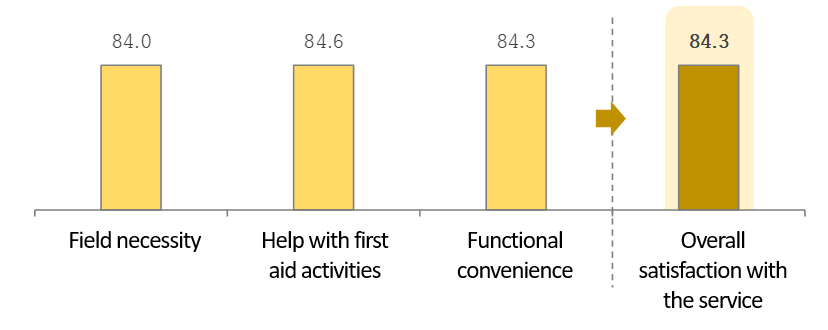 |  |  |
|  | |  |  | |
|  |  |  |  |  |


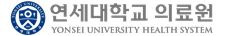


|  |  |
| --- | --- |

2. Patient class prediction service using AI

|  | ※ Patient class prediction service using AI  - This service uses real-time patient information as input to predict the treatment that should be performed on the patient at the hospital and presents the results to the pre-hospital providers as output. |
| --- | --- |

[ figure 2] Patient class prediction service using AI

|  | |  |  | |
| --- | --- | --- | --- | --- |
|  |  |  |  |  |
|  |  | (N=114) |  |  |
|  |  | 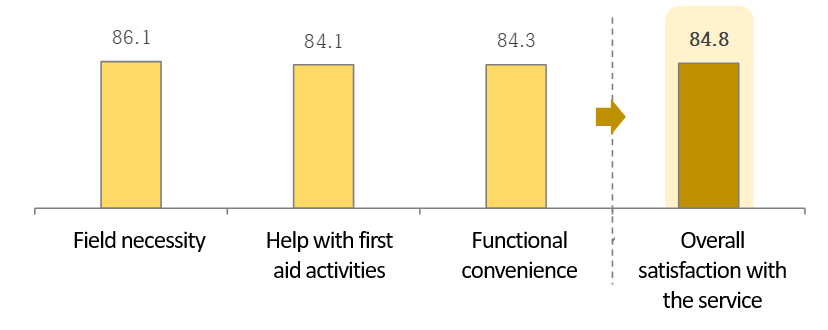 |  |  |
|  | |  |  | |
|  |  |  |  |  |


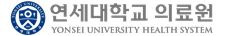


|  |  |
| --- | --- |

**3. Optimal Transfer Hospital Selection Service using AI**

|  | ※ Optimal Transfer Hospital Selection Service using AI  - This service provides pre-hospital providers with a real-time, prioritized list of hospitals that can provide the predicted care, and allows for simultaneous requests for acceptance to multiple hospitals. |
| --- | --- |

[ figure 3] Optimal Transfer Hospital Selection Service using AI

|  | |  |  | |
| --- | --- | --- | --- | --- |
|  |  |  |  |  |
|  |  | (N=114) |  |  |
|  |  | 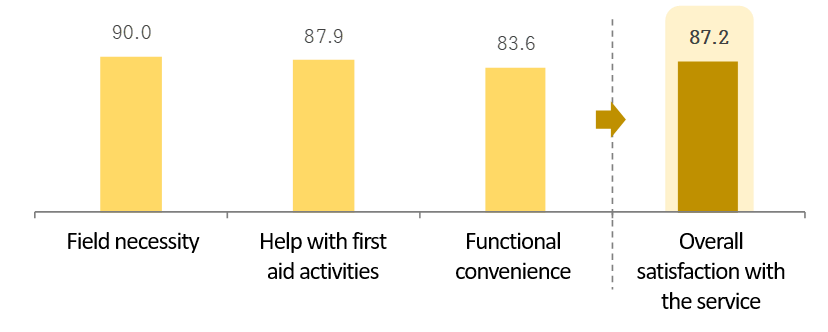 |  |  |
|  | |  |  | |
|  |  |  |  |  |


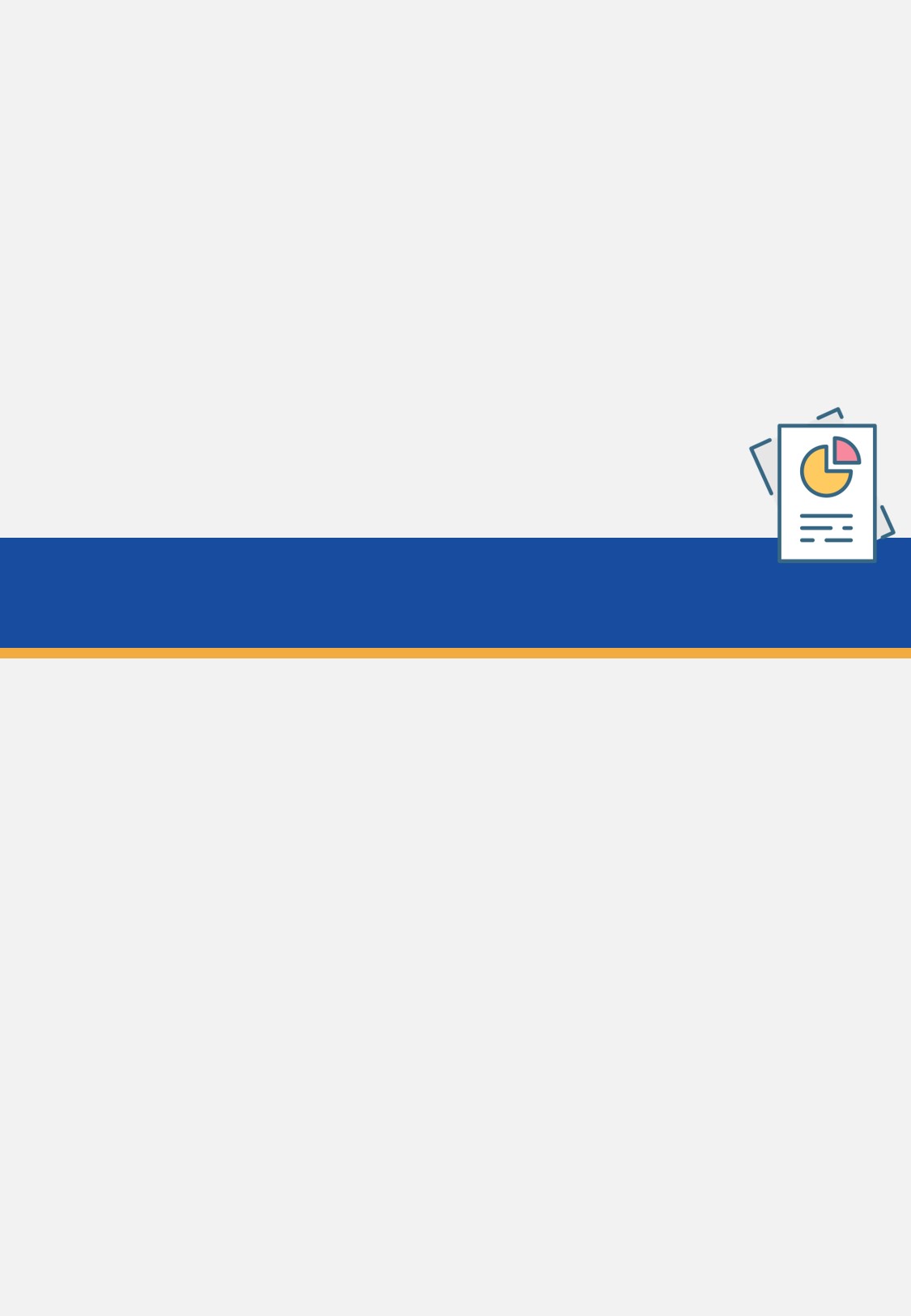


**CONNECT AI system satisfaction survey**

Appendix

1. Survey

| AI Guidance Service of Standardized protocol for first aid |
| --- |

**Q01. This is a question about the AI Guidance Service of Standardized protocol for first aid. Depending on your satisfaction, please respond among the views (7 points).**

| ※ If you agree to 7 points (Strongly agree), 4 points (Neutral), and 1 point (Strongly disagree), you can give me a higher score, and if you agree to a lower score, you can give me a lower score. |
| --- |
| ※ AI Guidance Service of Standardized protocol for first aid  - The EMS kiosk presents standardized first aid for pre-hospital providers to perform based on patient information entered in real-time through the CONNECT AI system. |

| **No** | **Evaluation Contents** | **Strongly disagree** | | **-----------** | | **Neutral** | | | **----------** | | **Strongly agree** | |
| --- | --- | --- | --- | --- | --- | --- | --- | --- | --- | --- | --- | --- |
| 1 | The AI Guidance Service of Standardized protocol for first aid is a necessary service in the field | ① | ② | | ③ | | ④ | ⑤ | | ⑥ | | ⑦ |
| 2 | The AI Guidance Service of Standardized protocol for first aid is helpful for first aid activities | ① | ② | | ③ | | ④ | ⑤ | | ⑥ | | ⑦ |
| 3 | The AI Guidance Service of Standardized protocol for first aid is conveniently configured | ① | ② | | ③ | | ④ | ⑤ | | ⑥ | | ⑦ |

| Patient class prediction service using AI |
| --- |

**Q02. This is a question about the Patient class prediction service using AI. Depending on your satisfaction, please respond among the views (7 points).**

| ※ If you agree to 7 points (Strongly agree), 4 points (Neutral), and 1 point (Strongly disagree), you can give me a higher score, and if you agree to a lower score, you can give me a lower score. |
| --- |
| ※ Patient class prediction service using AI  - This service uses real-time patient information as input to predict the treatment that should be performed on the patient at the hospital and presents the results to the pre-hospital providers as output. |

| **No** | **Evaluation Contents** | **Strongly disagree** | | **-----------** | | **Neutral** | | | **----------** | | **Strongly agree** | | |
| --- | --- | --- | --- | --- | --- | --- | --- | --- | --- | --- | --- | --- | --- |
| 1 | The Patient class prediction service using AI is a necessary service in the field | ① | ② | | ③ | | ④ | ⑤ | | ⑥ | | ⑦ | |
| 2 | The Patient class prediction service using AI is more conducive to first aid activities | ① | ② | | ③ | | ④ | ⑤ | | ⑥ | | ⑦ | |
| 3 | The Patient class prediction service using AI function is conveniently configured (convenient to use) | ① | ② | | ③ | | ④ | ⑤ | | ⑥ | | ⑦ | |
| Optimal Transfer Hospital Selection Service using AI | | | | | | | | | | | | |  |

**Q03. This is a question about the Optimal Transfer Hospital Selection Service using AI. Depending on your satisfaction, please respond among the views (7 points).**

| ※ If you agree to 7 points (Strongly agree), 4 points (Neutral), and 1 point (Strongly disagree), you can give me a higher score, and if you agree to a lower score, you can give me a lower score. |
| --- |
| ※ Optimal Transfer Hospital Selection Service using AI  - This service provides pre-hospital providers with a real-time, prioritized list of hospitals that can provide the predicted care, and allows for simultaneous requests for acceptance to multiple hospitals. |

| **No** | **Evaluation Contents** | **Strongly disagree** | | **-----------** | | **Neutral** | | | **----------** | | **Strongly agree** | |
| --- | --- | --- | --- | --- | --- | --- | --- | --- | --- | --- | --- | --- |
| 1 | The Optimal Transfer Hospital Selection Service using AI is a necessary service in the field | ① | ② | | ③ | | ④ | ⑤ | | ⑥ | | ⑦ |
| 2 | The Optimal Transfer Hospital Selection Service using AI is helpful for first aid activities | ① | ② | | ③ | | ④ | ⑤ | | ⑥ | | ⑦ |
| 3 | The Optimal Transfer Hospital Selection Service using AI is conveniently configured | ① | ② | | ③ | | ④ | ⑤ | | ⑥ | | ⑦ |
